# Physiological and Ultrastructural Alterations Linked to Intrinsic Mastication Inferiority of Segment Membranes in Satsuma Mandarin (*Citrus unshiu* Marc.) Fruits

**DOI:** 10.3390/plants11010039

**Published:** 2021-12-23

**Authors:** Xuefei Lian, Feifei Li, Yuanyuan Chang, Tie Zhou, Yuewen Chen, Tao Yin, Yunsong Li, Li Ye, Yan Jin, Xiaopeng Lu

**Affiliations:** 1 College of Horticulture, Hunan Agricultural University, Changsha 410128, China; 1753891547@stu.hunau.edu.cn (X.L.); changyuanyuan@stu.hunau.edu.cn (Y.C.); 8899@stu.hunau.edu.cn (T.Z.); chenyuewen@hunau.edu.cn (Y.C.); 2020-yintao@stu.hunau.edu.cn (T.Y.); tashanlys@stu.hunau.edu.cn (Y.L.); 18184246804@stu.hunau.edu.cn (L.Y.); jinyan@hunau.edu.cn (Y.J.); 2 National Centre for Citrus Improvement, Changsha 410128, China; 3 Institute of Horticulture, Hunan Academy of Agricultural Science, Changsha 410125, China; lifly607@163.com

**Keywords:** Satsuma mandarin, intrinsic mastication inferiority, segment membrane, pectin, gene expression

## Abstract

Chewing texture is important for fresh citrus fruits, and the mastication trait of a segment directly determines chewing texture. Roughing disorder impairs the quality of Satsuma mandarin fruits, and it is typically correlated with intrinsic mastication inferiority (IMI). This study explored the role of segment membranes (SMs) in IMI. Similar to IMI in roughing-disordered fruits, segment shear force significantly enhanced relative to controls (CK); cell layers and cell wall thickness increased also in inferior masticating SMs. The ‘Miyamoto Wase’ cultivar exhibited larger segment shear force and more SM cell layers than ‘Juxiangzao’. In SMs, vessel cells could be divided into outside layers where segments adjoin and inside layers where juice sacs grow from. The inside vessel cell layers in the inferior masticating SMs were denser. Vessels with a length of 200 to 300 μm and a diameter of 5 to 15 μm predominated in SMs. The average vessel diameter enlarged by 13% to 16.5% in inferior masticating SMs, depending on cultivars. Furthermore, there was a decrease in vessels with a diameter <5 μm and an increase in vessels >10 μm in the inferior masticating SMs. Between phenotypes, protopectin increased significantly throughout development of inferior masticating SMs, while water-soluble pectin increased during the later stages of development. In one inferior masticating SM sample, protopectin and water-soluble pectin levels were higher in the inner-ring area than those in the outer-ring area. Correspondingly, expression of *CuPME21* which is involved in pectin hydrolysis was consistently upregulated in the inferior masticating SMs throughout fruit development. The findings in this work provide novel insights into citrus SM structure and its IMI.

## 1. Introduction

Unlike citrus juice consumption, fresh citrus fruits are particularly popular in Asian countries. For fresh consumption, organoleptic and texture properties are important determinants of fruit quality, and chewiness is a direct quality concern for fresh citrus fruits. The chewiness property of citrus pulp was termed melting [1] or mastication [2] in previous publications. Intrinsic mastication inferiority (IMI), also termed inferior melting character or inferior mastication trait, is a standard quality determinant in citrus fruits, and it refers to lots of residues or rough fibres remaining after chewing fruit segments. IMI occurs in most citrus crops, especially Satsuma mandarin (*Citrus unshiu* Marc.). Segments are the main edible tissues of citrus fruits, and they are composed of the segment membranes (SMs) and juice sacs. SMs should be the most important factor determining the mastication trait of citrus [3,4].

SMs originate from the endocarp of citrus fruits, while the exocarp and mesocarp develop into the flavedo and albedo of peel, respectively [5,6]. In citrus fruits, segment membranes are composed of a few cell layers, and the cell wall composition and cell development associated with fruit mastication. Due to their impact on edibility, citrus SMs have been investigated widely, especially for Satsuma mandarins, in order to produce commercially attractive canned and peeled segments. Industrially, chemical processes involving acids and alkalis and pectic enzymes can be used to peel mandarin segments so they are ready to eat or for canning [7,8]. However, citrus SMs contain high levels of bioactive compounds and antioxidants, hence it is recommended to consume citrus fruits with edible tissues intact, rather than to consume juice or juice sacs alone [8,9]. Citrus SMs and extracts also promote gut health [10,11].

Segment shear force reflects the citrus mastication trait indirectly, and it can be employed as a quantitative profiler for segment mastication properties. For ‘Nanfeng’ tangerine, an SM thickness of 0.9 to 1.2 mm and a segment shear force of 1.15 to 1.64 kg segment^−1^ results in good segment mastication, but a thickness of 1.4 to 1.7 mm and a segment shear force of 2.63 to 5.70 kg segment^−1^ leads to IMI [4]. Fruit texture is associated with the composition of the cell wall [12,13,14]. Several reports showed that cell wall compositions including pectin, cellulose and lignin in fruit pulp are involved in citrus IMI. In Navel orange ‘Fengjie wancheng’ (*Citrus sinesis* Osbeck), more pulp protopectin, cellulose, hemicellulose and lignin but less water-soluble pectin resulted in tougher mastication relative to ‘Fengjie 72-1′ [1]. Levels of water-soluble pectin, protopectin and lignin in pulp contribute to the different mastication trait of three ‘Nanfeng’ tangerines [2]. In Kiyomi (*Citrus unshiu* Marcov × *Citrus sinensis* Osbeck) fruits, less cellulose, hemi-cellulose and lignin but more water-soluble pectin in SMs is crucial for efficient segment mastication; SM polygalacturonase and cellulase activities from 210 to 300 days after flowering are the main contributors to good mastication [15]. In contrast with the excellent mastication properties of ‘Shatangju’ (*Citrus reticulata* Blanco), the inferior mastication of ‘Nanfeng’ tangerine associates with high levels of cellulose and hemicellulose in SMs [3,16]. Based on analyses of six citrus cultivars, protopectin content in SMs but not water-soluble pectin is assessed to determine the mastication trait of citrus [17]. Compared with ‘Shiranui’ (*C. unshiu* Marcov. × *C. sinensis* Osbeck × *C. reticulata* Blanco), inferior mastication of ‘Kiyomi’ is linked with higher pectin and lignin levels but lower hemicellulose content in SMs. Moreover, the degree of pectin methyl esterification was lower for ‘Kiyomi’ SMs [18].

Roughing disorder is a comprehensive quality issue of citrus fruit, and it refers to aberrant fruit size, peel thickness and pulp texture. IMI is one of the typical phenotypes of roughing disorder, and it occurs universally in Satsuma mandarin fruits [19]. Roughing-disordered Satsuma mandarin exhibits visibly thickened and inferior masticating segments [20]. For ‘Nanfeng’ tangerine (*Citrus reticulata* Blanco cv. Kinokuni), fruits from a year-off tree showed roughing disorder characteristics including enlarged fruit size, increased SM thickness and IMI [21]. By contrast with ‘Nanfeng’ tangerine, IMI in roughing-disordered Satsuma mandarin is derived from an unbalanced carbohydrate distribution during fruit development.

In the present work, we comprehensively investigated physiological and ultrastructural alterations of the inferior masticating SMs. The ‘Juxiangzao’ cultivar, an early-ripening Satsuma mandarin selected from ‘Oita Wase’, displayed good mastication, but IMI still occurred in roughing-disordered fruit. We also assessed the main causes of IMI in ‘Miyamoto Wase’ SMs, another traditional early-ripening cultivar. The results revealed the changes of inferior masticating SMs in physiology, ultrastructure, and gene expression which provide novel insight into citrus IMI.

## 2. Results

### 2.1. Characteristics of Segment Membranes (SMs) in Roughing-Disordered Satsuma Mandarin Fruits

**Figure 1:**
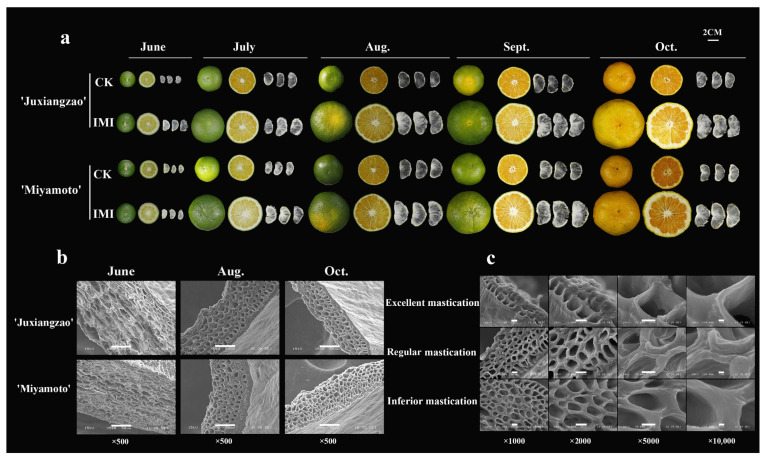
Characteristics of roughing disorder in Satsuma mandarin fruits and its intrinsic mastication inferiority (IMI) of segment membranes (SMs). (**a**) Development of SMs in roughing-disordered Satsuma mandarin fruits. (**b**) SMs in two Satsuma mandarins under scanning electron microscopy; the bar indicates 50 μm. (**c**) Ultrastructural morphology of SMs with different mastication degrees; the bars in pictures with ×1000, ×2000, ×5000 and ×10,000 magnifications indicate 10 μm, 10 μm, 5 μm and 1 μm respectively.

Significantly larger fruits with SMs severely thickened occurred in roughing-disordered fruits. SM transparency increased with citrus fruit development, consistent with changes of thickness. In June, roughing-disordered fruits displayed increments in fruit size, SM size and peel thickness relative to CK fruits, while SM transparency showed no significant differences between phenotypes. During July, ‘Juxiangzao’ SMs were more transparent in CK fruits than that in roughing-disordered fruits, but SMs of ‘Miyamoto Wase’ showed similar transparency between phenotypes. From July to maturity, SM transparency increased significantly in CK fruits but changed little in roughing-disordered fruits (Figure 1a). For CK fruits, no morphological differences were found between ‘Juxiangzao’ and ‘Miyamoto Wase’ SMs. For SMs, the circle centre area was always thicker than the external area. SM thickness in CK fruits decreased with fruit development, and the regular cell shape and arrangement were observed meanwhile (Figure 1b). Based on fruit chewing texture, SMs with different mastication trait were analysed. As expected, excellent mastication of SMs was correlated with thinner cell walls, while the inferior masticating SMs exhibited thicker cell walls. The increase in SM thickness originated from both cell layers and cell wall thickness (Figure 1c).

Segment shear force reflects the mastication trait of SMs indirectly. Consistent with transparency, the inferior masticating segments displayed larger shear force relative to CK. When CK segments were cross-cut, ‘Juxiangzao’ segments had a light shear force per segment of 1.85 to 4.82 kg during fruit development, compared with that of ‘Miyamoto Wase’ 3.06 to 7.04 kg per segment. During CK fruit development, segment shear force decreased continuously for ‘Juxiangzao’ after July, whereas for ‘Miyamoto Wase’ it peaked in August and then decreased. Between phenotypes, segment shear force was about two-fold higher in roughing-disordered fruits than that in CK fruits. In roughing-disordered fruits, ‘Juxiangzao’ segments were firmest in September while ‘Miyamoto Wase’ segments were firmest in August. When segments were length-cut, shear force showed about 50% decline relative to cross-cut. During fruit development, segment shear force in length-cut and cross-cut shared a change pattern, but the inferior masticating segments were always with the higher shear force (Table 1).

### 2.2. Ultrastructural Construction Alterations in the Inferior Masticating Segment Membranes

**Figure 2:**
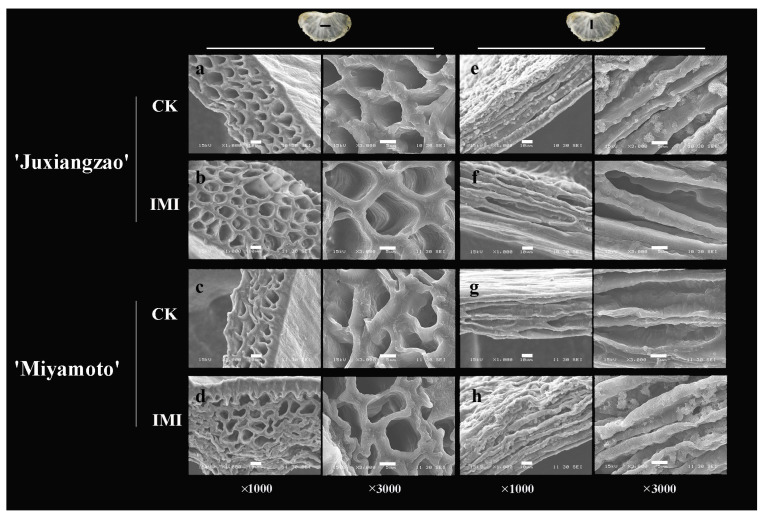
Ultrastructural morphology of inferior masticating SMs in ‘Juxiangzao’ and ‘Miyamoto Wase’. (**a**,**b**) cross section of CK and inferior masticating SMs in ‘Juxiangzao’. (**c**,**d**) cross section of CK and inferior masticating SMs in ‘Miyamoto Wase’. (**e**,**f**) Longitudinal section of CK and inferior masticating SMs in ‘Juxiangzao’. (**g**,**h**) Longitudinal section of CK and inferior masticating SMs in ‘Miyamoto Wase’. The bars in pictures with ×1000 and ×3000 magnifications indicate 10 μm and 5 μm, respectively.

Many ultrastructural changes were observed in the inferior masticating SMs. Between phenotypes, the inferior masticating SMs exposed more cell layers than CK SMs, resulting in thicker SMs finally. Individual cells developed vigorously in the inferior masticating SMs (Figure 2a–d). In longitudinal sections, vessels were connected to each other regularly. Vessels in SMs were arranged following the veins. In longitudinal sections, vigorous vessels could be identified also in the inferior masticating SMs (Figure 2e–h).

**Figure 3:**
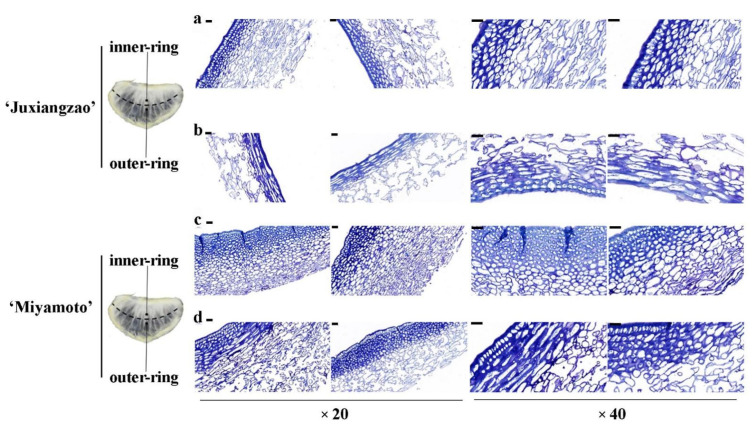
Anatomical differences between inner-ring and outer-ring parts in one inferior masticating SM. (**a**,**b**) Staining for the inner-ring and outer-ring cells of ‘Juxiangzao’. (**c**,**d**) Staining for the inner-ring and outer-ring cells of ‘Miyamoto Wase’. The bar indicates 50 μm.

Based on transparency, semicircles of SMs could be divided into outer- and inner-ring parts. Histochemical staining of the inferior masticating SMs showed that more cell layers in the inner-ring part caused aggravated thickness in both cultivars (Figure 3a,b). SM inside is defined as that on which juice sacs grow, while its outside is the one segments adjoin. Within one section plane, cells clearly differentiated into outside and inside layers. Inside cell layers were arranged more densely while outside cell layers were disaggregated in SMs. Among cultivars, ‘Miyamoto Wase’ displayed a denser cell arrangement with more cell layers than in ‘Juxiangzao’ in both outer- and inner-ring parts (Figure 3c,d).

Vessel morphology was also altered in the inferior masticating SMs. Vessel length extended in the inferior masticating SMs of ‘Juxiangzao’, but it did not differ between phenotypes of ‘Miyamoto Wase’. In more detail, vessel length in SMs ranged from 50 to 200 μm, with minimal differences between cultivars. Vessels with a length of 200 to 300 μm accounted for 70% to 80% of SMs, while a length of 150 to 200 μm accounted for just 15% to 20%. Less than 6% of SM vessels had a length <50 μm or >200 μm. For both cultivars, vessel length displayed the same distribution profile in CK and inferior masticating SMs. By contrast, vessel diameter significantly expanded in the inferior masticating SMs for both cultivars. About 95% to 97% of vessels had a diameter of 5 to 15 μm in both ‘Juxiangzao’ and ‘Miyamoto Wase’. Between phenotypes, the inferior masticating SMs contained more vessels with a diameter >10 μm but fewer vessels with a diameter <5 μm (Table 2).

### 2.3. Changes in Pectin, Cellulose and Lignin in the Inferior Masticating Segment Membranes

The inferior masticating SMs typically contained more pectins. During June, when fruits had just started to enlarge, SMs contained their highest protopectin content for both CK and roughing-disordered fruits. Afterwards, protopectin in SMs decreased with fruit development until fruit maturity. SMs of ‘Juxiangzao’ and ‘Miyamoto Wase’ showed similar protopectin accumulation patterns throughout fruit development (Table 3).

**Figure 4:**
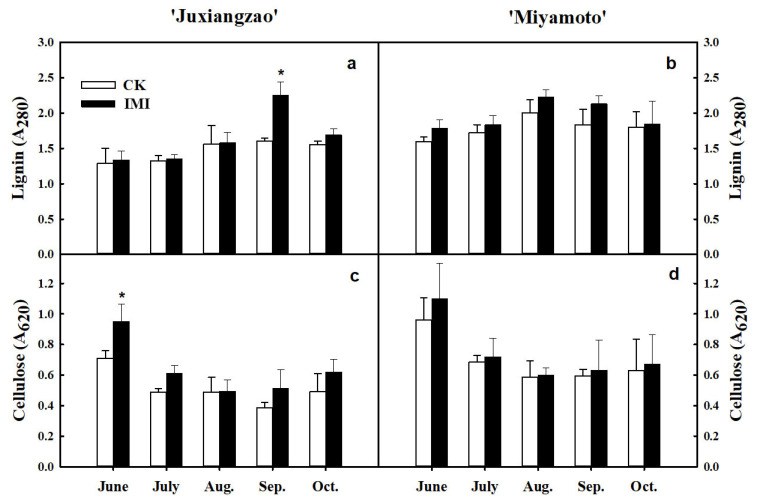
Lignin and cellulose accumulations in inferior masticating SMs of ‘Juxiangzao’ and ‘Miyamoto Wase’. (**a**,**b**) Lignin content of SMs in ‘Juxiangzao’ and ‘Miyamoto Wase’. (**c**,**d**) Cellulose content of SMs in ‘Juxiangzao’ and ‘Miyamoto Wase’. The data are presented as mean ± SD for three replicates. Asterisks (*) indicate significant difference relative to control (*p <* 0.05).

In both cultivars, a higher protopectin content in the inferior masticating SMs was observed from June, when roughing disorder was identified initially, and this difference was maintained during fruit development. In mature fruits, inferior masticating SMs of ‘Juxiangzao’ had a protopectin level ~37.5% higher than that of CK fruits, and the level in ‘Miyamoto Wase’ was 27.1% higher. Between cultivars, ‘Miyamoto Wase’ exhibited a higher SM protopectin level than ‘Juxiangzao’ in both CK and roughing-disordered fruits. When fruit ripened in October, SMs in CK fruits of ‘Miyamoto Wase’ had 18.1% higher protopectin content than that of ‘Juxiangzao’, and the difference was 9.5% higher in the inferior masticating SMs. For water-soluble pectin in both cultivars, the inferior masticating SMs displayed a higher content than CK SMs after August. Similar to protopectin accumulation, ‘Miyamoto Wase’ always displayed higher level of water-soluble pectin than ‘Juxiangzao’ in both CK and inferior masticating SMs (Table 3). Relative to CK, cellulose and lignin levels changed barely in the inferior masticating SMs, except timepoints during development (Figure 4a–d).

**Figure 5:**
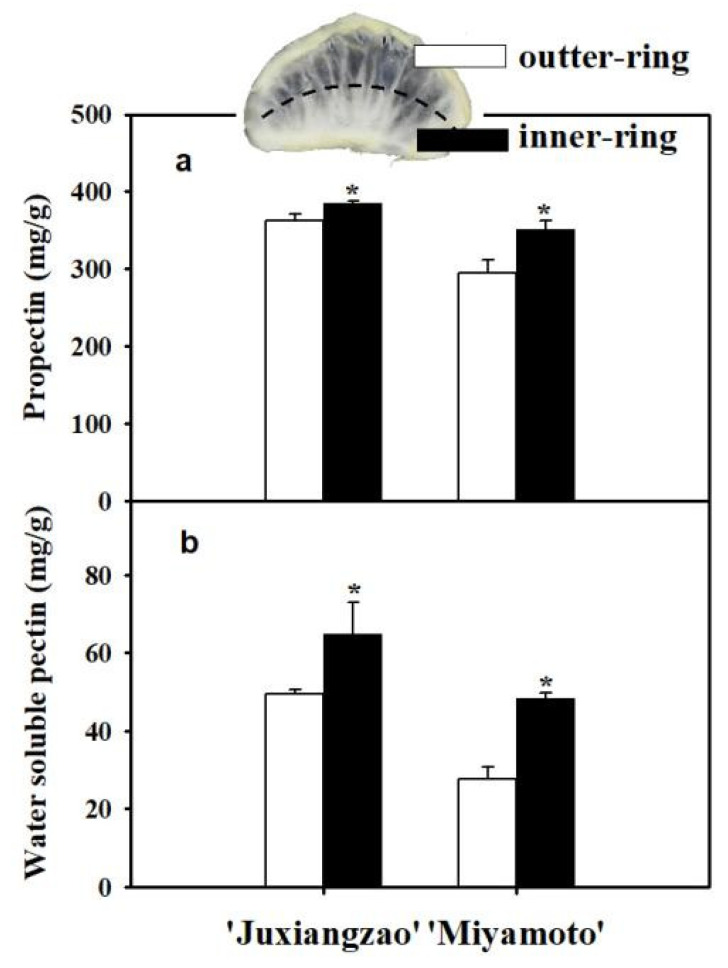
Protopectin and water-soluble pectin accumulations in inner- and outer-ring parts in one inferior masticating SM. (**a**) Protopectin difference between inner- and outer-ring parts. (**b**) Water-soluble pectin difference between inner- and outer-ring parts. The data are presented as mean ± SD for three replicates. Asterisks (*) indicate significant difference relative to control (*p <* 0.05).

In one inferior masticating SM sample, the pectin distribution differed between inner- and outer-ring parts. In accordance with visible transparency, poorer transparency correlated with higher protopectin and water-soluble pectin levels in the inner-ring part (Figure 5a,b).

### 2.4. Expression Patterns of Genes Involved in Pectin Metabolism in Segment Membranes

**Figure 6:**
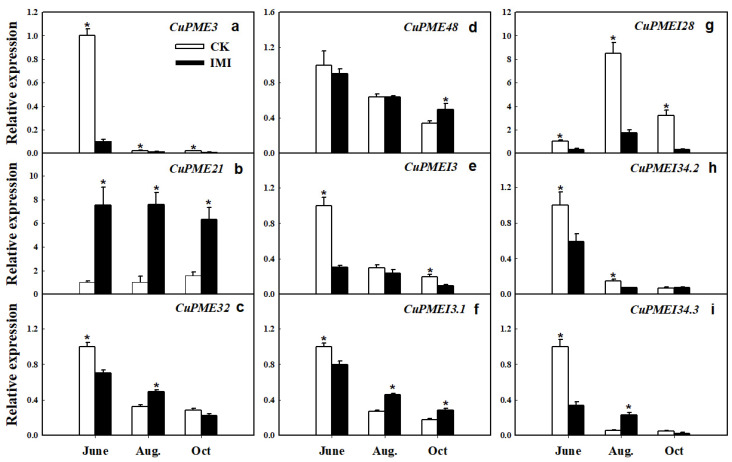
Gene expression patterns of *CuPME*s and *CuPMEI*s in CK and inferior masticating SMs. (**a**–**d**) Expression patterns of four *CuPME* genes. (**e**–**i**) Expression patterns of five *CuPMEI* genes. The data are presented as mean ± SD for three replicates. Asterisks (*) indicate significant difference relative to control (*p <* 0.05).

According to previous reports, 45 genes encoding pectin α-1,4-D-galacturonosyltransferase (GAUT), pectin methylesterase (PME), PME inhibitor (PMEI) and pectin lyase (PL) were found with expressions in SMs. Among these, 16 genes showed a two-fold change in expression in ‘Kiyomi’, which had a higher SM pectin level. Among the 16 differently expressed genes (DEGs), nine of them expressed in ‘Juxiangzao’ SMs, including four *CuPME*s and five *CuPMEI*s. Most of these nine genes expressed with highest levels at June, and then decreased progressively during SM development. *CuPME21* was consistently upregulated in the inferior masticating SMs throughout development, compared with CK SMs. Additionally, *CuPMEI3.1* was expressed significantly higher in the inferior masticating SMs from August, when SMs gradually became transparent. By contrast, *CuPME3* was downregulated in the inferior masticating SMs during June, and expression levels decreased further thereafter (Figure 6).

## 3. Discussion

For fresh citrus fruit, pulp chewing property is one of the important quality factors. IMI occurs in all citrus crops, but it is particularly prominent in easy-peeling mandarins such as Satsuma mandarin. Satsuma mandarins are universal in Asian countries, but their chewing properties are an ongoing issue. Fruit roughing disorder is a particular problem in Satsuma mandarins, and it is linked to IMI generally. In roughing-disordered fruits, vigorous SM development contributes to fruit IMI approximately. However, the mechanism by which SM development leads to IMI is poorly understood.

Inferior masticating SMs are characterised by a visible increase in thickness and a decrease in transparency. Based on histology, increased cell layers and cell wall thickness appear to be responsible for SM thickening and then IMI (Figure 1 and Figure 2). Differences between inner- and outer-ring parts were observed in one SM in this study. Increased thickness and inferior transparency of inner-ring SMs derived from more cell layers and a denser cell arrangement (Figure 3). Additionally, more protopectin and water-soluble pectin accumulated, presumably causing IMI of SMs.

IMI differed between citrus fruits depending on genetic or physiological factors. Typically, ‘Nanfeng’ tangerines are renowned for IMI. Based on texture analysis, IMI of ‘Nanfeng’ tangerines was quantified firstly by an SM thickness >1.4 mm and a shear force >2.63 kg per segment [4]. Both ‘Juxiangzao’ and ‘Miyamoto Wase’ are early-ripening Satsuma mandarins, and their inferior masticating fruits had a length-cut shear force of 3.3 and 2.4 kg per segment, respectively, 1.5- to 2.8-fold higher than that in CK fruits (Table 1). With segment shear force in accordance with the mastication trait, both cultivars occurred IMI, in which ‘Juxiangzao’ shows better fruit mastication than ‘Miyamoto Wase’. Histochemical analysis indicated thinner SMs of ‘Juxiangzao’ resulting from fewer cell layers and a looser cell arrangement (Figure 2 and Figure 3). These results imply that ‘Juxiangzao’ is superior in terms of SM development and mastication, while ‘Miyamoto Wase’ is likely to display IMI easily. Vessel diameter altered significantly in the inferior masticating SMs (Figure 1c and Figure 2). A diameter of 5 to 10 μm was observed in all SMs, but an increase in vessels with a diameter >10 μm and a decrease in vessels with a diameter <5 μm probably contributed to IMI through which carbohydrate flux increased.

Pectin, lignin, cellulose and hemi-cellulose are all believed to contribute to the mastication trait of citrus. Comparison of six ‘Nanfeng’ tangerines suggested that fruit mastication is determined by water-soluble pectin, protopectin and lignin levels, but not cellulose or hemicellulose; low *PG* and *PME* expression is consistent with high pectin content [2]. Flesh melting of peach differs from mastication in citrus fruits, but both are related to fruit pectin metabolism. Peach fruits are classified into melting flesh and non-melting flesh [22,23,24], and changes of *PG* in gene expression and gene structure are responsible for peach melting [25,26]. In the present work, cellulose and lignin accumulation did not alter in the inferior masticating SMs for both ‘Juxiangzao’ and ‘Miyamoto Wase’ (Figure 4a–d). However, significantly more protopectin and water-soluble pectin were observed in the inferior masticating SMs of both cultivars (Table 3). In one inferior masticating SM, the inner-ring part showed much tougher masticating than the outer-ring part, as well as higher protopectin and water-soluble pectin levels in both cultivars (Figure 5a,b). These results probably suggest pectin contributes specifically to the inferior masticating SMs in roughing-disordered Satsuma mandarin fruits.

A few publications reported genes involved in citrus fruit mastication, in which gene expression was studied most using fruit pulp containing SMs and juice sacs. For instance, the ‘Fengjiewancheng’ cultivar with inferior mastication properties had higher levels of pulp cellulose and protopectin, corresponding to lower mRNA levels of *PG*, *PME* and *Cel* at harvest time [1]. Similar research from this research group on ‘Nanfeng’ tangerines indicated that pectin could be a major contributor to citrus mastication trait, and *PG* and *PME* played important roles in this process [2]. Recently, comparative RNA-sequencing was performed on SMs, and the results showed that *CuGAUT*, *CuPME*, *CuPMEI* and *CuPL* genes in pectin pathways are associated with citrus fruit mastication [18]. Together with previous findings, our current results suggest that expression of *CuPME21* which is involved in pectin hydrolysis linked to citrus IMI. Expression of *CuPME21* probably indicates IMI of SMs at an early development stage.

## 4. Materials and Methods

### 4.1. Plant Materials

Eight-year-old ‘Juxiangzao’ (*Citrus unshiu* Marc.) trees at the Experiment Station of Hunan Agricultural University, Changsha, Hunan province, and 10-year-old ‘Miyamoto Wase’ (*Citrus unshiu* Marc.) trees planted in Shaoyang, Hunan province, were used for the study of SMs. All trees were grafted on trifoliate orange (*Poncitrus trifoliata* (L.) Raf.) rootstocks. Due to some roughing-disordered fruits in one tree, a total of 30 uniform trees containing triplicate groups for each cultivar were selected for sampling. Roughing-disordered fruits just growing on the top of the canopy were sampled for IMI, and regular fruits growing on the downward canopy served as controls (CK). Ten fruits from each replicate group were collected in the middle of June, July, August, September and October. Representative fruits were photographed with a Canon EOS 6D camera (Canon, Japan). Three of the 10 fruits were used for texture analysis, SM preparation, and FAA fixation in formalin: glacial acetic acid:70% alcohol (5:5:95). The isolated SMs from other fruits were stored at −40 °C for biochemical analysis and −80 °C for gene expression analysis.

Additionally, ‘Miyamoto Wase’ (*Citrus unshiu* Marc.) planting in Dongjiang lake district, Zixing, Hunan province is known well for its excellent fruit mastication. Hence, the SMs of that were used also for ultrastructural comparison, and ‘Juxiangzao’ SMs from CK and IMI served as regular and inferior mastication respectively. All these three SM types were sampled at the same time in the mature season.

### 4.2. Determination of Segment Firmness

For segment shear force measurement, six segments with relatively uniform size in one fruit were selected and three of them were cut transversely and another three were cut longitudinally. The central area of a segment was cut to perform this test. Three biological replicates were undertaken for each sample. Fruit segment shear force was measured using a TA.XT Plus texture analyzer (Stable Micro Systems, London, UK) and the data were analysed by the Texture Exponent 32 (Stable Micro Systems, London, UK) software accompanying the instrument. An HDP/BS test probe was employed with a speed of 1 mm·s^−1^ before measurement, 3 mm·s^−1^ during and after measurement, a measurement distance of 20 mm, and a trigger value of 5 g [4].

### 4.3. Microscopic Morphology

To reveal the ultrastructure of SMs, a central area 3 mm in length and 1 mm in width was isolated and examined with a JSM-6380LV scanning electron microscope (SEM; Jeol, Tokyo, Japan) as described previously [20]. Three biological replicates were performed for each part of the scanning electron microscope, and representative images were selected.

Paraffin sections were prepared to examine the cell arrangement in SMs. An inferior masticating SM film was divided into inner-ring and outer-ring parts. Central areas 5 mm in length and 3 mm in width were isolated from each ring. All samples were dehydrated until transparent, immersed in wax, and embedded in paraffin. Sections made from embedded samples were stained by Toluidine Blue and imaged by a Panoramic DESK scanning instrument (3DHISTECH, Hungary). Representative images were obtained using a CaseViewer software (3DHISTECH, Hungary). Three biological replicates were performed for each sample.

### 4.4. Vessel Isolation and Statistics

A 1 cm^2^ piece of film isolated from SM in matured fruits was used to separate vessel cells according to the description [27] with a slight modification. Samples were immersed in Jeffery separation solution (10% nitric acid:10% chromic acid, 1:1) for 7 to 8 h then rinsed with water. Using an NIB610 inverted fluorescence microscope (Yongxin Optical, Ningbo, China), 100 vessels were selected randomly for image collection using ScopeImage 9.0 image processing software (Yongxin Optical, Ningbo, China). The length and diameter of the vessels were measured according to the image corresponding to the scale size using ImageJ Plus6.0 software (National Institutes of Health, Bethesda, MD, USA). The vessel separation and molecule statistics were calculated for the three fruit groups.

### 4.5. Determinations of Pectin, Cellulose and Lignin

SMs were dried and ground into powder firstly. Protopectin and water-soluble pectin were quantified by carbazole colorimetry according to the instructions of the Soluble Pectin and Original Pectin Kit (Keming Biotech, Jiangsu, China).

Cellulose in SMs were analyzed according to the descriptions of Bu et al. [28]. An accurate measure of 1 g of SM powder was added to 3.0 mL of acetic/nitric reagent (80% acetic acid:concentrated nitric acid, 10:1 *v*/*v*), heated in a boiling water bath at 100 °C for 30 min, centrifuged for 30 min at 4 °C and 10,000× *g*, and then the supernatant was discarded. The residue was washed with distilled water twice. The washed residue dissolving in 6 mL of 67% sulfuric acid was shaken well for 1 h, and then 0.1 mL of the solution was pipetted into a glass test tube, where it was mixed with 4 mL of deionised water and 10 mL of cold anthrone reagent (0.2 g anthrone in 100 mL concentrated sulfuric acid). The samples were placed in a boiling water bath for 10 min and then cooled to room temperature. The absorbance of sample was measured at 620 nm using a NanoPhotometer P-Class USB ultraviolet spectrophotometer (Implen GmbH, Munich, Germany). Each determination was performed for three replicates.

Lignin in SMs were analyzed according to the descriptions of Deng et al. [29]. Accurate 1 g of SM powder was added to 5 mL 95% ice-cold ethanol solution and then centrifuged at 4 °C for 20 min at 10,000× *g*. The collected pellet was washed three times with 3 mL 95% ethanol, and then washed with ethanol:hexane = 1:2 (*v*/*v*) three times. Pellets were collected for drying and transferred to tubes, after which 3 mL of 25% bromized acetyl–acetic acid was added. Then a water bath at 70 °C was used to incubate the tubes for 30 min before the reaction was stopped with the addition of 0.9 mL 2 M NaOH. The tubes were centrifuged at 4 °C for 20 min at 10,000× *g* after mixing with 5.1 mL ice acetic acid. Finally, we added 1 mL of supernatant to 9.0 mL of distilled water. The absorbance was measured at 280 nm using a NanoPhotometer P-Class USB ultraviolet spectrophotometer (Implen GmbH, Munich, Germany). Each determination was performed for three replicates.

### 4.6. Gene Identifications and Quantitative Polymerase Chain Reaction (qPCR) Analysis

According to published RNA-sequencing data from ‘Kiyomi’ and ‘Shiranui’ SMs, 45 genes associated with the pectin pathway encoding pectin α-1,4-D-galacturonosyltransferase (GAUT), pectin methylesterase (PME), PME inhibitor (PMEI) and pectin lyase (PL) were found to be expressed. Among these, 16 genes showing a two-fold change in expression were selected for further investigation [18]. Specific primers for qPCR are listed in Table S1. From the isolated SMs, an RNAprep Pure Plant Kit (Tiangen, Beijing, China) was employed to extract total RNA, which was specifically designed for materials rich in polysaccharides and polyphenols. RNA quality was determined by agarose gel electrophoresis and qualified with a NanoPhotometer P-Class ultraviolet spectrophotometer (Implen GmbH, Munich, Germany). The cDNA was synthesised using *Evo M-MLV* Reverse Transcription Reagent Master Mix (Aikeri, Hunan, China). A 10 μL reaction mixture containing 400 ng RNA and 2 μL 5× *Evo M-MLV* RT Master Mix was made up to volume with RNase-free H_2_O. Quantitative PCR was performed using a Talent Fluorescence Quantitative Detection Kit (Tiangen, China) and a CFX96 real-time PCR Detection System (Bio-Rad, Hercules, CA, USA). The *β-Actin* is selected as the reference gene. We prepared 10 μL reaction mixture containing 2× Talent qPCR Premix enzyme (5 μL), primers (0.8 μL), diluted template (1 μL) and ddH_2_O (3.2 μL). Thermal cycling involved pre-denaturation at 95 °C for 3 min, followed by 40 cycles of denaturation at 95 °C for 5 s, annealing at 58 °C for 10 s, and extension at 72 °C for 15 s. Expression of each gene was assessed using three biological replicates. Data were obtained using Bio-Rad CFX Manager 3.1 software. Relative gene expression was calculated using the CFX96 software. Three biological replicates were performed for each sample.

### 4.7. Statistical Analysis

Three biological replicates were conducted for all determinations and statistical calculations. Data were analysed statistically using Duncan’s multiple range tests in the analysis of variance (ANOVA) program of SAS (Cary, Raleigh, NC, USA), with statistical significance considered at *p* < 0.05.

## 5. Conclusions

Intrinsic mastication inferiority (IMI) occurs generally in roughing-disordered Satsuma mandarin fruits, and SMs play important roles in this process. Roughing-disordered fruits had firmer segments and thicker SMs, corresponding to more SM cell layers and thicker cell walls. A severe increase in cell layers correlates with the intrinsic mastication inferiority of citrus SMs. The ‘Miyamoto Wase’ cultivar showed larger segment shear force and more cell layers in SMs relative to ‘Juxiangzao’. An increase in vessels with a diameter of >10 μm and a decrease in vessels with a diameter of <5 μm correlated with IMI of SMs in roughing-disordered Satsuma mandarin fruits. Accumulation of protopectin and water-soluble pectin was also associated with IMI of SMs. Expression of *CuPME21* in SM was closely linked with IMI.

## Figures and Tables

**Table 1:** Segment shear force in CK and inferior masticating segments (kg segment^−1^). The data are presented as mean ± SD for three replicates.

		‘Juxiangzao’		‘Miyamoto Wase’	
		CK	IMI	CK	IMI
Cross-cut	July	4.82 ± 0.65	7.31 ± 0.90 *	5.04 ± 0.58	9.31 ± 0.67 *
	Aug.	2.19 ± 0.22	5.25 ± 0.66 *	7.04 ± 0.89	10.81 ± 0.67 *
	Sep.	2.65 ± 0.29	8.11 ± 0.40 *	3.66 ± 0.13	5.78 ± 1.23 *
	Oct.	1.85 ± 0.10	7.65 ± 0.87 *	3.60 ± 0.11	5.29 ± 0.57 *
Length-cut	July	2.64 ± 0.14	4.01 ± 0.20 *	2.68 ± 0.27	4.42 ± 0.12 *
	Aug.	1.62 ± 0.08	3.61 ± 0.76 *	3.80 ± 0.25	3.59 ± 0.67 *
	Sep.	1.29 ± 0.13	3.73 ± 0.37 *	1.78 ± 0.12	2.58 ± 0.29 *
	Oct.	1.19 ± 0.18	3.30 ± 0.43 *	1.59 ± 0.19	2.40 ± 0.36 *

Asterisks (*) indicate significant difference relative to control (*p <* 0.05).

**Table 2:** Morphologic statistics for vessel molecules in SMs. The data are presented as mean ± SD for three replicates or percent to 300 vessels.

		‘Juxiangzao’		‘Miyamoto Wase’	
		CK	IMI	CK	IMI
Average vessel length/μm		107.2 ± 3.6	127.7 ± 1.1 *	125.4 ± 10.8	118.6 ± 8.5
Average vessel diameter/μm		7.7 ± 0.3	8.7 ± 0.4 *	7.9 ± 0.5	9.2 ± 0.1 *
Vessel length	<50 μm	0.7%	0.7%	ND	1.0%
	50–100 μm	37.7%	26.3%	34.7%	36.0%
	100–150 μm	41.3%	44.3%	39.3%	43.3%
	150–200 μm	15.0%	24.7%	20.0%	14.7%
	>200 μm	5.3%	4.0%	6.0%	5.0%
Vessel diameter	<5 μm	5.3%	1.0%	4.3%	1.7%
	5–10 μm	79.3%	80.3%	79.0%	72.3%
	10–15 μm	15.3%	17.3%	16.3%	23.7%
	>15 μm	ND	1.3%	0.3%	2.3%

Asterisks (*) indicate significant difference relative to control (*p* < 0.05). ND indicates not detected.

**Table 3:** Protopectin and water-soluble pectin contents in SMs (mg g^−1^). The data are presented as mean ± SD for three replicates.

		‘Juxiangzao’		‘Miyamoto Wase’	
		CK	IMI	CK	IMI
Protopectin	June	425.69 ± 6.48	512.85 ± 7.85 *	269.60 ± 21.79	362.47 ± 17.13 *
	July	370.99 ± 40.43	543.47 ± 32.81 *	265.61 ± 6.74	319.99 ± 5.81 *
	August	345.46 ± 21.79	431.78 ± 35.74 *	367.44 ± 14.85	413.12 ± 11.66 *
	September	265.17 ± 0.91	298.89 ± 4.46 *	267.63 ± 20.03	287.82 ± 3.36
	October	168.60 ± 4.84	231.34 ± 4.37 *	199.06 ± 7.15	253.32 ± 24.58 *
Water-soluble pectin	June	14.57 ± 4.24	12.65 ± 1.88	16.04 ± 1.13	17.82 ± 2.11
	July	14.34 ± 1.93	15.92 ± 4.08	10.68 ± 0.48	10.00 ± 0.32
	August	12.33 ± 2.60	18.91 ± 3.15 *	14.75 ± 1.59	20.40 ± 2.19 *
	September	28.02 ± 4.29	24.82 ± 8.31	11.56 ± 0.33	32.85 ± 0.72 *
	October	14.63 ± 1.62	25.55 ± 4.44 *	17.95 ± 2.84	32.73 ± 3.17 *

Asterisks (*) indicate significant difference relative to control (*p <* 0.05).

## Data Availability

All data generated or analysed during this study are included in this published article.

## Supplementary Materials

The following supporting information can be downloaded at: https://www.mdpi.com/article/10.3390/plants11010039/s1, Table S1: Primers used in qPCR.
