# Supplementary material for: Physiological and Ultrastructural Alterations Linked to Intrinsic Mastication Inferiority of Segment Membranes in Satsuma Mandarin (Citrus unshiu Marc.) Fruits
Source: Plants (Basel). 2021 Dec 23;11(1):39. doi: 10.3390/plants11010039 (PMC8747345; doi:10.3390/plants11010039)
Supplement: Supplementary file 1 [file plants-11-00039-s001.zip › plants-1500904-supplementary.pdf]

Table S1. Primers used in qPCR.

Gene name	Gene ID	Description	Primer sequence (5′ to 3′)
CuPME3	orange1.1g008261 m.g	Pectin methylesterase	Forward：TTTACCACAATGGCACAACCC<br>Reverse：TTCTTCTTCGGCACCTCCACA
CuPME32	orange1.1g010718 m.g	Pectin methylesterase	Forward：TTTCTTGGTCAGTGCGGTTTC<br>Reverse：ATGAAAGAGGAGGCTGGCACT
CuPME21	orange1.1g039111 m.g	Pectin methylesterase	Forward：AACCGTTTGCCAACCCACAGA<br>Reverse：GGAAACTGTCGCCACAACCAC
CuPME48	orange1.1g039631 m.g	Pectin methylesterase	Forward：TAGCATACCTCAAGGGAACACC<br>Reverse：TGTTCCCTTGAGGTATGCTATT
CsPMEI34.2	orange1.1g029436 m.g	Pectin methylesterase inhibitor	Forward：GCCCAATCCGCAAAGTCATT<br>Reverse：CCCATCTCATCTAAGCAGTCG
CuPMEI28	orange1.1g038081 m.g	Pectin methylesterase inhibitor	Forward：GGCATTCCACTCCCGCAAACT<br>Reverse：CAGCCAAGTAAAGGTGGAAGA
CuPMEI34.3	orange1.1g027094 m.g	Pectin methylesterase inhibitor	Forward：AGACTTGGGTTAGTGCTGCTT<br>Reverse：AGGCTTCATTCCAGGGCTTTT
CuPMEI3.1	orange1.1g029211 m.g	Pectin methylesterase inhibitor	Forward：GAATAACTTCGTCCGAGATGC<br>Reverse：TTGTCACCGAGACGCCAGCAC
CuPMEI3	orange1.1g040679 m.g	Pectin methylesterase inhibitor	Forward：GCCTCCGCACGCTATCGTCCTA<br>Reverse：ACCGTGACCGTGCTTGCTTCC
β-Actin	orange1.1g032498 m.g	--	Forward: TGACTGATGAGAACTGCCAGAAG<br>Reverse：CCAATTCTCTCTTGAACCTGTCCTT
